# Sphingolipid Players in Multiple Sclerosis: Their Influence on the Initiation and Course of the Disease

**DOI:** 10.3390/ijms23105330

**Published:** 2022-05-10

**Authors:** Maria Podbielska, Toshio Ariga, Anna Pokryszko-Dragan

**Affiliations:** 1 Department of Neuroscience and Regenerative Medicine, Medical College of Georgia, Augusta University, Augusta, GA 30912, USA; toshio.ariga@yahoo.com; 2 Laboratory of Microbiome Immunobiology, Ludwik Hirszfeld Institute of Immunology & Experimental Therapy, Polish Academy of Sciences, 53-114 Wroclaw, Poland; 3 Department of Neurology, Wroclaw Medical University, 50-556 Wroclaw, Poland; anna.pokryszko-dragan@umed.wroc.pl

**Keywords:** antibody, central nervous system, ganglioside, lipid rafts, inflammation, MS therapy, MS mechanism, multiple sclerosis, neurodegeneration, neurological disease

## Abstract

Sphingolipids (SLs) play a significant role in the nervous system, as major components of the myelin sheath, contributors to lipid raft formation that organize intracellular processes, as well as active mediators of transport, signaling and the survival of neurons and glial cells. Alterations in SL metabolism and content are observed in the course of central nervous system diseases, including multiple sclerosis (MS). In this review, we summarize the current evidence from studies on SLs (particularly gangliosides), which may shed new light upon processes underlying the MS background. The relevant aspects of these studies include alterations of the SL profile in MS, the role of antibodies against SLs and complexes of SL-ligand-invariant NKT cells in the autoimmune response as the core pathomechanism in MS. The contribution of lipid-raft-associated SLs and SL-laden extracellular vesicles to the disease etiology is also discussed. These findings may have diagnostic implications, with SLs and anti-SL antibodies as potential markers of MS activity and progression. Intriguing prospects of novel therapeutic options in MS are associated with SL potential for myelin repair and neuroprotective effects, which have not been yet addressed by the available treatment strategies. Overall, all these concepts are promising and encourage the further development of SL-based studies in the field of MS.

## 1. Introduction

Sphingolipids (SLs) are important components of lipid bilayers that play a substantial role in the determination of cellular membrane properties and their dynamic changes. They also contribute to segregation within the membrane and the formation of its cholesterol and SL-enriched microdomains, defined as lipid rafts [1,2]. These microdomains are crucial for the compartmentalization of cellular processes, including intracellular cytoskeleton organization, transport and signaling, as well as interactions with the extracellular environment (e.g., exo- and endocytosis, ion channel function) [3]. Furthermore, SLs are associated with the dynamic balance between processes of cellular viability (growth and proliferation) and apoptosis [4]. All these unique properties of SLs are particularly relevant for the role that they play in the functions of the central nervous system (CNS). SLs are abundant within neuronal membranes and constitute a major component of the myelin sheath, indispensable for appropriate axonal conduction and their trophic supply. Changes in specific SL composition during the development of the CNS and differences in SL profiles throughout the CNS areas reflect the relevance of their regulatory and modulatory function [5,6,7]. Thus, it is not surprising that alterations in SL metabolism and content are often observed in the course of CNS diseases [8,9,10].

Multiple sclerosis (MS) is a disease associated with long-lasting injury disseminated throughout brain and spinal cord. Clinical manifestations of MS comprise a variety of symptoms and signs of neurological deficit, initially with fluctuating intensity (relapsing–remitting course, RRMS), but further also accumulating and resulting in multi-dimensional disability (primary/secondary progressive course, PPMS/SPMS). The background of MS is complex and involves interrelated processes of immune-mediated inflammation and neurodegeneration [11,12]. Originally, MS was recognized as a demyelinating disorder, with multifocal destruction of the myelin sheath as the main hallmark [13]. These lesions—especially in the paranodal (PN) areas—substantially affect the properties of the CNS myelinated axons, which is reflected in the specificity of clinical symptoms. Due to the loss of structural protection and trophic support, the axons become more vulnerable to various kinds of external stimuli; fast saltatory conduction of action potentials is impaired and additionally disturbed by the increased excitability of neurons [14]. Further research on the MS background revealed that the core pathomechanism of demyelination is associated with a dysregulated immune response, with a contribution of interacting genetic predisposition and several environmental factors, including exposure to sunlight, levels of vitamin D3, gut microbiota activity and recently highlighted Epstein–Barr virus infection [15,16,17,18]. Autoreactive immunocompetent cells (mainly CD4^+^ T helper, Th) reach the CNS due to disruption of the blood–brain barrier (BBB) and take part in the inflammatory cascade, targeting myelin antigens, which is mediated by cyto- and chemokines, as well as the humoral response driven by B cells [19,20,21]. At the same time, a slowly expanding neurodegenerative process develops, which involves ion channel dysfunction, oxidative stress and peroxysomal/mitochondrial dysfunction, resulting in an energetic deficit, and ultimately leads to axonal loss.

Despite significant progress in the understanding of the nature of MS in the last decade, as well as the increasing availability of diagnostic and therapeutic options, there is still ongoing extensive research in this field, especially with regard to aspects that have not been fully elucidated or sufficiently addressed. The studies on de- and remyelination have focused mainly on proteins as targets of autoimmune attack or mediators of inflammatory activity and the role of lipids in the background of MS, and the diverse course of the disease has only recently gained more attention [15,22,23]. Considering the properties of SLs and gangliosides and their role in the CNS, they seem a promising subject of investigation. In this review, we present the current evidence from studies on SLs (particularly gangliosides), which may shed new light upon the processes underlying MS’ pathophysiology, with possible diagnostic and therapeutic implications.

## 2. Role of SLs in the CNS

### 2.1. SLs as Components of the CNS Myelin Sheath

#### 2.1.1. Myelin Architecture

**Figure 1:**
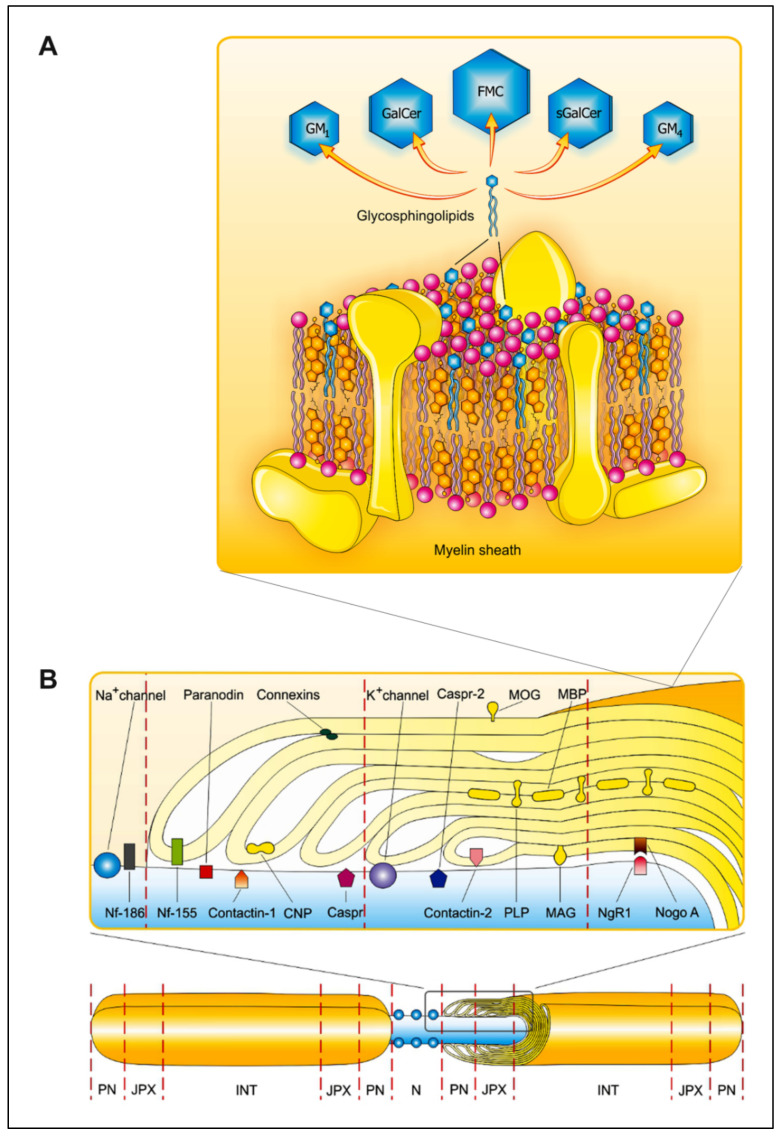
Composition of the myelin sheath in the central nervous system. (**A**) The diagram presents the distribution of the major myelin components, specifically complex lipids that comprise cholesterol, phospholipids and glycosphingolipids (GSLs), as well as proteins in the CNS. GSLs include the following compounds: fast migrating cerebrosides (FMCs), galactosylceramide (GalCer), mono-sialoganglioside (GM1), sialosyl-galactosylceramide (GM4) and sulfatide (sGalCer). Lipids such as cholesterol, PLs and GalCer remain in a relatively constant molar ratio, which is 2:2:1. The myelin components are indicated as follows: cholesterol in orange, PLs in pink, GSLs in blue and proteins in yellow. (**B**) Myelinated regions of axon are interspersed with non-myelinated ones, designated as nodes of Ranvier (N). Three domains can be distinguished within myelinated regions: paranode (PN), juxtaparanode (JXP) and internode (INT). Distribution of the proteins characteristic for these domains, along with Na and K channels, is also displayed. Abbreviations: Caspr—Contactin-associated protein, CNP—2′3′-cyclic-nucleotide 3′-phospodiesterase, NgR1—Nogo-receptor 1, Nogo A—neurite outgrowth inhibitor A, MAG—myelin-associated glycoprotein, MBP—myelin basic protein, MOG—myelin oligodendrocyte glycoprotein, Nf-155—Neurofascin 155, Nf-186—Neurofascin 186, PLP—proteolipid protein. Adapted from reference [28] and modified.

**Figure 2:**
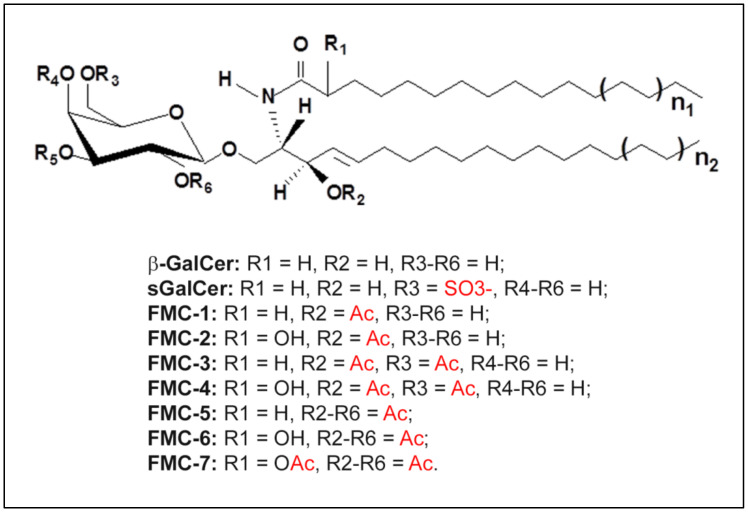
Structure of β-GalCer and its derivatives. Diagram shows a basic glycosphingolipid structure where the R groups are positioned and a relevant substitution results in β-GalCer, sGalCer as well as fast migrating cerebrosides (FMC-1, FMC-2, FMC-3, FMC-4, FMC-5, FMC-6 and FMC-7), respectively. Ac—acetyl group. Adapted from reference [31] and modified.

**Figure 3:**
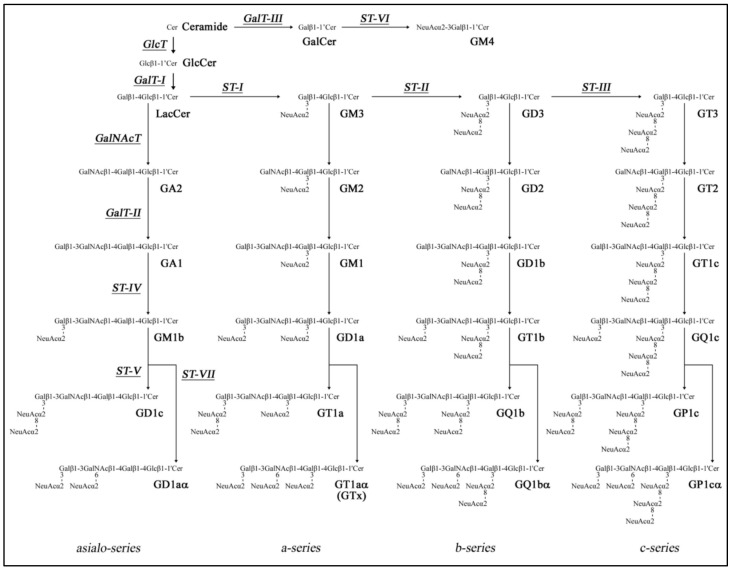
Ganglioside metabolic pathways. The nomenclature is based on Svennerholm [32] and IUPAC-IUBMB Joint Commission on Biochemical Nomenclature [33]. Glycosyltransferases that catalyze the synthesis of GSLs have been underlined. The following gangliosides—GD1aα, GT1aα, GQ1bα and GP1cα—belong to the α-series gangliosides [34]. Abbreviations: Cer—ceramide, Gal—galactose, GalNAc—N-acetylgalactosamine, GalNAc-T—N-acetylgalactosaminyltransferase I (GA2/GM2/GD2/GT2-synthase, GalT-I—galactosyltransferase I (lactosylceramide synthase), GalT-II—galactosyltransferase II (GA1/GM1/GD1b/GT1c-synthase), GalT-III—galactosyltransferase III (galactosylceramide synthase), Glc—glucose, GlcT—glucosyltransferase (glucosylceramide synthase), LacCer—lactosylceramide, NeuAc—N-acetylneuraminic acid, ST-1—sialyltransferase I (GM3-synthase), ST-II—sialyltransferase II (GD3-synthase), ST-III—sialyltransferase III (GT3-synthase), ST-IV—sialyltransferase IV (GM1b/GD1a/GT1b/GQ1c-synthase), ST-V—sialyltransferase V (GD1c/GT1a/GQ1b/GP1c-synthase), ST-VII—sialyltransferase VII (GD1α/GT1aα/GQ1bα/GP1cα-synthase). Adapted from reference [35].

The CNS myelin sheath comprises multiple spiral layers formed by oligodendrocytes, ensuring the isolation and structural strengthening of axons [24]. The predominant myelin constituents are depicted in Figure 1. A characteristic feature of this multilayer membrane is the high content of lipids, which constitute approximately 80%. The remaining percentage belongs to proteins. The lipids within the CNS myelin include phospholipids and glycosphingolipids (GSLs), with the only representative of sterols being cholesterol (Figure 1A). The PLs are represented by phosphatidylethanolamine, phosphatidylserine, phosphatidylcholine, phosphatidylinositol and sphingomyelin (SM). Among GSLs, galactosylceramide (GalCer) is the most abundant component and accounts for 32% of the CNS myelin lipid content. Other GalCer derivatives include sulfated and acetylated derivatives represented by sulfatides (sGalCer) [25] and fast migrating cerebrosides (FMCs) [26], respectively (Figure 2). The more complex derivatives are characterized by sialylated ones, namely N-acetylneuraminic acid-containing GSLs (Figure 3)—especially mono-sialogangliosides such as GM1 and GM4 [27] (Figure 1A).

Apart from protective and supportive functions, the myelin sheath facilitates fast saltatory conduction within axons, due to Ranvier nodes (Figure 1B) (see Section 2.1.2).

#### 2.1.2. Myelin Sheath Organization in the CNS

Nodes of Ranvier, small unmyelinated axonal domains, are characteristic elements in the myelin sheath in the CNS [29]. The ability of these nodes to propagate fast and effectively action potentials along the axons depends on their specialized molecular organization (Figure 1B). The nodal domain is flanked by the PNs, formed by the interaction between the axolemma and distal, uncompacted loops of myelin. The PN loops generate a specialized junction with the axon, often referred to as the axoglial junction. The axoglial junction, which comprises interacting axonal (Contactin and Contactin-associated protein, Caspr) and glial (Neurofascin 155, Nf-155) components, is required to establish an effective PN diffusion barrier [30]. The absence or dysfunction of any of these components results in impairment of the axoglial junction. This diffusion barrier, which separates the nodal Na channels from the K channels localized in the adjacent juxtaparanode (JPX) region, is then no longer effective. The majority of the axon comprises the internodes (INTs), which are located beneath the compact myelin sheath. The nodal complex, which consists of Na channels and cell adhesion molecules, is anchored to the cytoskeleton and linked with extracellular matrix proteins, which provides its stability. Na channels associate with Caspr-2 and the glycosylphosphatidylinositol-anchored TAG-1 (Contacin-2), which can be found within axonal and glial components.

Gangliosides play a substantial role in the organization of nodes and the PNs in myelinated fibers. They provide proper compartmentalization of adhesion molecules (Nf-155 and Contactin/Caspr), essential elements of the PN cytoarchitecture [36]. Furthermore, associated gangliosides contribute to the appropriate compartmentalization of Kv1 channels and anchoring proteins (Caspr-2, Contacin-2) in JXP regions [37].

### 2.2. Physiological and Immunological Features of SLs in the CNS

SLs play very important roles in the vital functions of the CNS cells [3]. They participate in the formation and adequate functioning of the CNS myelin sheath. Therefore, their altered metabolism leads to the decompaction and destabilization of the myelin structure [38]. However, SLs not only contribute to myelin formation and the maintenance of its stability, but also act as bioactive modulators of many processes [39], i.e., the proliferation, growth and survival of cells [40,41,42]. Myelin and oligodendrocytes enriched in SM, SL and cholesterol lipid microdomains [43] have been considered to play significant roles associated with signal transduction and the trafficking of proteins [44,45].

#### 2.2.1. Sialic-Acid-Containing SLs Properties and Functions in the CNS

Gangliosides, sialic0acid-containing GSLs (Figure 3) primarily located on the plasma membranes, are particularly abundant in the nervous system. The distribution of specific gangliosides in different types of the CNS cells and particular brain areas has not yet been comprehensively investigated. Early studies reported the abundance of gangliosides mainly in myelin and in neuronal cells [46]. Myelin contains high amounts of GM1 and GM4 (Figure 1A), while GM1, GM4 and GD3 can be detected in adult oligodendrocytes [47]. Conversely to neurons, astrocytes produce mainly GM3 and GD3, with lower amounts of GM4. There is less evidence of gangliosides’ presence in microglia, but these cells are suggested to display GM1 and significant amounts of GD3, when stimulated by pro-inflammatory mediators [48]. Remarkably, gangliosides account for 80% of all glycans and more than 75% of the sialic acid present in the brain. Gangliosides are relevant contributors to the maturation and stability of the nervous tissue. They are important players both in the development of neurons and the compartmentalization of their membrane domains, which is reflected in their concentration at the growing tips of neurites [49].

Gangliosides are known to participate in cell–cell recognition, interaction and adhesion, as well as in several aspects of malignant transformation and cancer metastasis [50,51]. Being important constituents of lipid rafts, gangliosides are implicated in the modulation of signal transduction and provoke an immune response [34,50,52]. These SLs containing N-acetylneuraminic acid possess many immunomodulatory functions that include the suppression of lymphoproliferation and regulation of production of cytokines [53].

Gangliosides, similarly to other SLs, are also known to be immunogens. Recent reports in the literature describing high levels of antibodies against gangliosides in the sera of individuals with Guillain Barré syndrome and other immune-mediated polyneuropathies indicate their role in the background of these disorders [54]. They were also suggested as pathogenic factors for several other neuroinflammatory and neurodegenerative diseases [55,56,57], including MS (see Section 3.4).

In addition to the above-described functions, both physiological and pathological levels of gangliosides can play a significant role in maintaining neuronal Ca^2+^ homeostasis and in the regulation of Ca^2+^ signaling, acting as modulators of ion channels and transporters’ activity [58]. Ceramide (Cer) and carbohydrate structures are supposed to be crucial for gangliosides’ biological activity, including their impact on synapse transmission and regeneration [59,60], and potentially neuroprotective effects [61].

Gangliosides can also work as co-receptors [62]. A study on the α2,3-sialyllactose moiety of GM3 and GM1, located within lipid rafts, revealed its properties as a low-affinity receptor for soluble α-Klotho [63], a protein required for the integrity and protection of neurons, hypothesized to prevent accelerated aging and cognitive decline [64].

#### 2.2.2. Role of Extracellular-Vesicle-Associated SLs in the CNS

Extracellular vesicles (EVs), released within the CNS or migrating through the BBB, have recently gained a great deal of attention in respect to the CNS physiological function and regulation of the immune response [65,66,67]. These nanosized particles, including exosomes and microvesicles (MVs), could play a substantial role in intercellular communication by transporting a large variety of biologically active molecules, i.e., SL species [68].

EVs impact recipient cell function and are supposed to contribute to the pathophysiology of CNS diseases. SL constituents of EVs may play a relevant role as effective carriers of a variety of active molecules, as well as engaging directly in the initiation or modulation of neuroinflammatory and neurodegenerative processes [69].

EV-associated SLs’ signatures are considered as promising biomarkers for the detection and monitoring of CNS disorders as well as potential therapeutic targets (see Section 3.7).

## 3. Role of SLs in MS Pathology

### 3.1. Ganglioside Alterations in MS

Current studies indicate that alterations in ganglioside metabolic pathways or the proportion of their particular components could appear in many neurodegenerative disorders, e.g., Alzheimer’s disease (AD) [70], amyotrophic lateral sclerosis [71], Huntington’s disease [72] and Parkinson’s disease [73]. Such observations have been made also in MS [74], whose pathology combines immune-mediated inflammation and degenerative processes. Table 1 displays the ganglioside alterations in MS. Yu et al. first characterized gangliosides isolated from the unaffected white matter (WM) and demyelinating plaques in brain tissue specimens from patients with MS [75]. Compared to WM, the plaques showed a decrease in GM1 and GM2, and complete loss of GM4. Most of the plaques had a significant elevation of GD2 and GD3 as well as the slower migrating polysialogangliosides. The increase in GD3 was of particular interest as it was attributed to reactive astrocytosis occurring in the plaque tissues.

Marconi et al. reported an immunohistochemical analysis of ganglioside expression in MS brains [76]. In the WM of the normal CNS (nCNS), GM1 and GD1b were found on astrocytes, whereas GD1a and GD2 were found on oligodendrocyte precursors and mature oligodendrocytes, respectively. In the grey matter (GM) of the nCNS, only GM1 and GD2, as well as GT3 and its acetylated derivatives, were detected on the neuronal cells. Interestingly, in chronic MS lesions, the astrocytic gangliosides GM1 and GD1b were preferentially expressed on oligodendrocyte precursors. Instead, selective expression of GT1b was observed within plaques—the astrocyte and oligodendrocyte precursors—but not in other neurological diseases (ONDs).

Examination of spinal cord specimens from patients with MS indicated decreased amounts of GM4, GM1, GD1b and GQ1b in comparison to tissues from the nCNS [77]. However, GM3 and GD3 were highly increased with respect to the nCNS. A slight elevation of GM2 and a slight reduction in GT1b were also observed. Zaprianova et al. reported ganglioside patterns in a chronic relapsing experimental autoimmune encephalomyelitis (EAE) model [78]. Significant increases in GM1 and GD1a were captured in the brain and spinal cord tissues. These changes found in the brains of patients with MS are consistent with demyelination.

Analysis of a pooled MS sample of cerebrospinal fluid (CSF) indicated increases in GM1 and GM3 in comparison to non-MS controls [79]. Consistent with these findings was the report by Miyatani et al., who demonstrated increased levels of GM1 in the CSF of 16% of examined patients with MS with respect to OND [80]. The authors also observed an increased concentration of GD3 in 23% of patients with MS, in which 8% of them showed a dramatic increase in sulfated glucuronyl paragloboside. These abnormalities can reveal the pathological features attributed to MS plaques, such as demyelination and gliosis.

Regarding the plasma of patients with MS, earlier studies showed that the total ganglioside concentration was significantly lower than that in healthy subjects (HS) [81]. In contrast, Sela et al. reported a significantly increased ganglioside concentration in the sera of patients with MS [82]. A detailed study of the ganglioside profile in plasma revealed that GM3 and GD3 were slightly elevated in patients with MS. In turn, Zaprianova et al. reported significant increases in GM1 and GD1a and a decreased level of GM3 in the sera of patients with the first clinical episode suggesting MS [83]. The patients with subsequent MS relapses (especially those with a long duration of the disease) presented a significant decrease in GM1 and an increased level of GD1a in the serum. The increase in GD1a seems to indicate the presence of neurodegeneration already at the onset of MS, continuing over the course of the disease. On the other hand, an increase in GM1, the main myelin ganglioside, is apparently a marker of inflammatory demyelination, most active at the early stage of MS [83]. Analysis of peripheral blood lymphocytes in patients with RRMS in the remission phase indicated an increased level of total gangliosides in 39% of MS patients examined in comparison to HS [82].

### 3.2. Alterations of Other SL Metabolism in MS

The CNS SL composition has been extensively studied in MS in order to detect lipid signatures in the pathogenesis of the disease (Table 2). In particular, the SL content of WM, GM and both active (Ac-MS) and inactive (In-MS) plaques in different stages of MS was analyzed in great detail to discern a primary lipid defect. An early report from 1968 indicated a reduced GalCer/sGalCer ratio in the altered WM of patients with MS [84]. Many years later, in 2000, Marbois at al. demonstrated reduced sGalCer content in the altered WM and MS lesions in comparison to the nCNS. In addition, there were significant increases in the amount of hydroxylation of sGalCer (h24:0-sGalCer) in MS plaques in relation to the nCNS [85]. In turn, Singh et al. found Cer content to be increased in areas around MS plaques compared to the X-adrenoleukodystrophy brain [86].

Later, a great deal of progress regarding the analysis of MS-related lesions in the CNS tissues was made. Analysis of the altered WM in Ac-MS and In-MS indicated that the level of specific SLs, e.g., C18:0-Cer, C20:0-Cer, C22:0-SM and C24:0-SM, was below the level found in NAWM of the nCNS. In turn, studies of the altered GM of Ac-MS and In-MS revealed that the level of C20:0-Cer, C22:0-Cer and C16:0-SM was lower than that observed in the nCNS [87]. Qin et al. reported that in WM and plaques from the brains of patients with MS, sphingosine (Sph) and C16:0/C18:0-Cer levels were increased while the sphingosine 1-phosphate (S1P) level was decreased as compared with matched controls or pathological autopsy specimens of AD [88]. In addition, C18:0-Cer was found to be accumulated in reactive astrocytes of Ac-MS lesions. Furthermore, mass spectrometry results confirmed the upregulation of C16:0-, C18:0- and C20-Cer subspecies during demyelination in an in vivo model. They also found the level of Sph to be increased and SIP to be decreased [89]. We have recently reported distinctive SL modification patterns occurring in chronic MS lesions [90]. We noted that the level of major dihydroceramide (dhCer) subspecies in Ac-MS lesions was higher than that observed in NAWM of the nCNS. Sphingolipidomic analysis indicated different alterations in In-MS. We observed reduced levels of Cer and its precursors (dhCer and SM), while the levels of hexosylceramide (HexCer) and Cer 1-phosphate (C1P) were significantly elevated—in both cases, in relation to NAWM of the nCNS and Ac-MS. Our findings indicate that SL metabolic pathways in the advanced stages of MS may differ, depending on the presence of inflammatory activity (active versus inactive plaques).

Recent evidence suggests that an impaired SL pathway may reflect MS activity and progression. Specifically, such an association has been shown for perturbation in Cer metabolism, a precursor of complex SLs. Checa et al. reported that a significantly increased level of C16:0-HexCer correlated well with the Expanded Disability Status Scale (EDSS) in both RRMS and progressive MS subtypes [93]. Concerning the role of other complex SLs, Mayo et al. reported that the lactosylceramide (LacCer) level and, associated with its synthesis, β-1,4-galactosyltransferase 6 (B4GALT6) expression were increased in MS lesions [91].

Cer and its glycosylated derivatives were also examined in other biological specimens, such as the CSF [92,93,94], serum [95], plasma [96,97,98] and white blood cells (WBCs) [97] of patients with MS. It was reported that the elevated concentrations of C16:0-Cer, C24:0-Cer and C16:0-HexCer found in the CSF of MS patients were able to impair mitochondrial function, followed by axonal injury [92]. The presence of C16:0-HexCer in the CSF of patients with MS was also confirmed by Checa et al. [93]. In contrast to reduced sGalCer levels in MS tissues [85], Haghighi et al. found increased levels of sGalCer in the CSF of patients with MS [94]. Moreover, Moyano et al. reported that the level of C18:0- and C24:1-sGalCer found in plasma in patients with RRMS correlated well with their disability level, assessed with EDSS [96]. Interestingly, Kurz et al. observed the levels of C16-Cer, C24:1-Cer, C16-GluCer, C24:1-GluCer and C16-LacCer in plasma to be altered. The same group also reported that C24-Cer and C16-LacCer subspecies found in WBCs were reduced [97]. In line with these discoveries, Filippatou et al. showed significant changes for specific SLs in the serum of MS patients, as compared to HS. They included alterations of dhCer, Cer, HexCer and LacCer subspecies [95]. Moreover, some of the aforementioned subspecies (dhCer, Cer, HexCer and LacCer) were altered only in patients with progressive MS [95]. In addition, Amatruda et al. identified lipids related to faster clinical deterioration in the plasma of patients with PPMS [98]. Thus, C14:0-SM, C20:0-HexCer and C18:2-lysophosphatidic acid (LPA) seem promising candidates for markers of immune-mediated inflammation and progression in MS, which deserve further exploration.

### 3.3. The Role of the CNS Lipid-Raft-Associated SLs in the Pathogenesis of MS

The altered composition of lipid rafts in the nervous tissue may contribute to the pathogenesis of CNS disorders, including MS [100]. These alterations were suggested to modulate signal transduction, adversely affect the function of neurons and result in the death of glial cells; all these aspects are important in the pathology of MS.

It was reported that alteration of Nf-15, a protein that is associated with lipid rafts [101,102,103], may be involved in the pathogenesis of MS. This cell adhesion molecule, representing the L1 family, is specifically colocalized at the PN (Figure 1B) [104] and plays an important role in the stabilization of the PN structure, necessary for the maintenance of myelin sheath integrity [105]. Nf-155 is expressed specifically in myelinating glia [106] and represents a sensitive marker of inflammation and myelin damage [107]. Antibodies against this protein inhibited axonal conduction in a complement-dependent manner. The distribution of this protein is altered in MS, which results in damage to myelin and the emergence of axonal dysfunction [101,105]. Schafer et al. reported that mice lacking Cer galactosyltransferase, an enzyme that is required for the production of GalCer and sGalCer, exhibited an altered structure of the PN loops, as well as myelin structural and functional abnormalities [103]. Immunohistochemical staining of the nodes of Ranvier from sciatic nerves and optic nerves revealed that Nf-155 was dramatically reduced in mutant mice. Compared to the wild type, in lipid rafts isolated from the brain membrane homogenates of mutant mice, there was a remarkable reduction in the amount of Nf-155. Thus, it can be supposed that the loss of myelin-associated GalCer may alter the PN lipid raft composition. These results suggest that the reduction in lipid-raft-associated Nf-155 is a consequence of the perturbation of myelin lipids and that Nf-155 is essentially required for PN formation and maintenance. Similar PN abnormalities were found in the brains of GM2-synthase-deficient mice, which lacked all major brain gangliosides but expressed GM3 and GD3 [37]. These mutant mice revealed disruption of the PN junctions and altered localization and function of ion channels, resulting in slowing motor nerve conduction. Immunostaining near the nodes of Ranvier in these mice revealed that Nf-155 staining was partially decreased. Likewise, the amount of Nf-155 in cerebral membrane homogenates was reduced in these mice in comparison with wild-type ones. Thus, the loss of complex gangliosides caused disruption of the PN junctions and resulted in a reduction in lipid-raft-associated Nf-155. These findings indicate an important role of complex gangliosides in stabilizing interactions between neurons and glia at the PN junctions in peripheral nerves. Furthermore, Zhang et al. examined the lipid raft components in rat brains after perinatal hypoxic–ischemic damage and reported an association with the expression of Nf-155 and GM1 in lipid rafts [102]. Interestingly, after the administration of GM1, there was an increase in GM1 content in lipid rafts, resulting in the concomitant expression of Nf-155. GM1 may promote the repair of the structure of lipid rafts, enable their association with Nf-155, stabilize the PN structure and thus prevent myelin sheath damage, revealing also neuroprotective properties. Overall, reduced lipid raft association of Nf-155 in Ac-MS lesions is linked with the disassembly of the PN junction, which may contribute to demyelination as the main feature of MS pathology [101].

### 3.4. Antibodies against SLs in MS: Potential Candidate Biomarkers of Pathophysiology with Clinical Utility

Increasing interest in the role of anti-lipid antibodies in MS has been noted recently [15]. Initially, anti-ganglioside antibodies were detected in the serum and the CSF of patients with immune-mediated polyneuropathies [54,108]. A number of antibodies against gangliosides were found in the serum and the CSF of patients with MS (Table 3). Screening of serum antibodies for their reactivity against gangliosides, performed independently in different laboratories, indicated reactivity against GM1 in the range between 20% up to 38% of MS patients examined [109,110,111,112,113,114]. Only Koutsouraski et al. detected serum anti-GM1 reactivity in a much higher percentage (approximately two-fold) of MS patients [115]. Zaprianova et al. suggested that IgG anti-GM1 antibodies, found in the serum of patients with RRMS, could be involved in demyelination. Moreover, the authors concluded that these antibodies were associated with the clinical progression of MS [114], but not with brain atrophy [112]. Increased serum anti-GM1 reactivity occurred along with reactivities against asialo-GM1 [110,113], GD1a [113,116], GD1b [115] and GQ1b [115] in some patients with MS.

GM3, as the major ganglioside representative, was detected in the plasma of MS patients but also HS. It was reported that anti-GM3 antibodies were found more frequently in the sera of patients with PPMS and SPMS subtypes than in RRMS [117].

Interestingly, anti-GD2-like IgM autoantibodies were found in the sera of 30% of MS patients, with approximately 10% also reacting with GD3 and/or GD1a [118]. Moreover, anti-GD2-like IgM reactivity correlated positively with the degree of disability [118].

Besides serum anti-ganglioside antibodies, their presence was also detected in the CSF of patients with MS. Mata et al. found elevated titers of IgG antibodies against GD1a [116], while Kasai et al. observed increased levels of IgG against GM4 [119].

According to the current view on the MS background, neurodegeneration constitutes an important component, besides inflammatory demyelination [120]. Emerging axonal damage is regarded as the main pathological substrate for the progression of disability in the course of disease [121]. Although it is not clear whether anti-ganglioside antibodies contribute to axonal degeneration or appear as a consequence, they might be considered as a marker of this process. Several studies showed the potential of anti-ganglioside antibodies to cause BBB disruption [122], as well as conduction block at the level of the neuromuscular junction, and to prevent axonal regeneration [120,123]. Interestingly, Ravindranath et al. reported that the impact of anti-ganglioside IgM antibodies upon the BBB leakage is concentration-dependent but complement-independent [124].

For a long time, myelin lipid antigens have been overlooked as potential targets for immune-mediated attack in MS. However, there is increasing evidence that antibodies not only against gangliosides (Table 3) but also against GalCer [119,125], acetylated GalCer [126], sGalCer [127,128] and their complexes [129], PLs [130] as well as oxidized PLs, oxidized sterols and SM [129,131,132], can be specifically found in the body fluids of patients with MS. Moreover, anti-ganglioside antibodies were considered as potentially useful indicators in the differentiation of MS from other mimicking disorders [133].

In summary, anti-ganglioside antibodies could be candidate biomarkers for particular stages or types of MS as well as a measurement for MS activity and/or progression. The insight into immunoglobulins as indicators of CNS inflammation and neuronal/axonal damage could offer intriguing prospects for the pathophysiology of MS. It has not been clarified whether the B cell response initiates elements of MS pathogenesis or develops secondary to the Th1-mediated activity [99]. However, identifying SL antigen(s) that are specific to MS (or its particular phases) may appear relevant for a better understanding of the processes underlying the MS background [134], with potential diagnostic and therapeutic applications [15].

### 3.5. Gangliosides’ Effect upon Cellular Response in MS

The autoimmune response targeting the CNS myelin components, i.e., the proteolipids and GSLs, along with an impaired balance between pro-inflammatory (Th1, Th17) and anti-inflammatory (Th2) cytokines, is considered to be a crucial mechanism beyond MS initiation [135]. Gangliosides are supposed to play a relevant role in the initiation and modulation of this inflammatory process. Monteiro de Castro G et al. reported the effects of gangliosides on shifting Th1 to Th2/Th3 cytokine profiles during the acute phase of EAE [53]. In a group of Lewis rats treated with gangliosides, low expression of IFN-γ mRNA and high expression of TGF-β mRNA were observed, resulting in mild disease symptoms.

Shamshiev et al. [136] demonstrated that T cells recognizing self-glycolipids are more frequent in MS patients than in HS. They suggested that gangliosides derived from myelin could be presented together with CD1 molecules by macrophages and astrocytes, and thus become an additional target of the inflammatory cascade, initiated by autoreactive T cells.

It was also suggested that a ganglioside-targeted cellular response may contribute not only to the destruction of myelin, but also to axonal damage. Pender et al. [137] indicated increased levels of auto-reactive T cells against GM3 and GQ1b in patients with PPMS. These autoreactive circulating lymphocytes were found more often in MS patients, as compared to HS or ONDs. The authors speculated that this activity could be involved in axonal loss, predominating in progressive forms of MS [137].

### 3.6. SL Ligands for Receptors Expressed on NKT Cells and Their Modulatory Functions in MS

SLs may serve as ligands for receptors expressed on natural killer T (NKT) cells. There has recently been growing interest in invariant NKT (iNKT) cells, an innate type of CD4^+^ lymphocytes having an invariant T cell receptor (iTCR). The characteristic feature of this population is its reactivity with lipid antigens presented by the CD1 molecule [138]. These cells are also able to respond to α-GalCer, a synthetic GSL derived from the marine sponge *Agelas mauritianus* [139]. Besides synthetic GSL, α-GalCer and its analogues, the still growing list of lipids that are stimulants of iNKT cells includes endogenous antigens (e.g., iGB3, FMCs) as well as exogenous bacterial ligands.

NKT cells have been proven to display a regulatory function towards the autoimmune response, involved in the background of many diseases, including MS [138]. An emerging body of evidence points to the modulatory role of SL ligands for their receptors in this process. In some reports, α-GalCer has been shown to prevent EAE [140,141,142,143,144]. Singh et al. suggested that such a protective effect of repeated α-GalCer injections might be due to Th2 stimulation [141]. However, Pal et al. observed no effect upon EAE after repeated α-GalCer injections [143], and Jahng et al. indicated a protective effect from EAE using a single administration of α-GalCer only [140]. Moreover, Furlan et al. indicated the suppression of EAE activity after the administration of α-GalCer along with complete Freund’s adjuvant, but only when given subcutaneously and not intraperitoneally [144]. The authors concluded that the critical factor to obtain the protective effect of α-GalCer is the administration route.

In order to better define the impact of NKT cells upon MS, O’Keeffe et al. conducted a quantitative analysis of iNKT cells in the peripheral blood of patients with MS. They indicated an increased percentage of T cells expressing the iTCR, defining the iNKT cells, in patients with MS in comparison to HS [145]. To clarify the relevance of these findings, further functional studies were performed [31]. A mixture of endogenous myelin-derived acetylated β-GalCer antigens (FMC-5 and FMC-7) and purified FMC-7 (Figure 2) was used as ligands to stimulate iNKT from patients with MS in vitro. These cells failed to respond, or indicated hyporesponsiveness, to the stimulation of these ligands, contrary to the findings among HS controls, including the excessive production of cytokines by Th1, Th2 and Th17 cells [31]. A similar phenomenon was observed in the case of iNKT cells from patients with MS stimulated with synthetic α-GalCer antigen, with the exception of some for TNF-α production. The state of GSL ligand-driven anergy may have significant clinical implications [146].

### 3.7. The Role of EV-Associated SLs in MS

Keeping in mind the role of lipid homeostasis in the adequate functioning of the CNS, it seems likely that SL-laden EVs may influence the development and progression of MS. Increased levels of EVs in both the plasma [147] and the CSF [148] of MS patients seem to support this hypothesis. However, more studies are warranted to determine whether, for example, C16:0-sGalCer associated with EVs isolated from plasma can be used as a biomarker of progression in MS [147]. It was shown that the injection of MVs from microglial cells into the brain in vivo resulted in enhanced inflammation and caused disease exacerbation [149]. Microglia-derived MVs were also shown to influence synaptic activity through enhanced SL metabolism, specifically Cer and Sph production, in neurons [150]. In line with these facts is also our recent observation that Cer-laden exosomes (specifically C16:0-, C24:0- and C24:1-Cer) released from human oligodendroglioma cells, followed by exposure to TNF-α and IFN-γ in vitro, caused the death of these cells in a time-dependent manner [151]. We speculated that these EVs can exert a similar function in vivo and promote the autoimmune response that causes demyelination in the CNS. Thus, modulation of the EV lipidome may represent another promising therapeutic target.

## 4. A Future Prospect for MS Therapy Based on SL Investigations

Currently available therapeutic strategies in MS include pulses of corticosteroids applied in acute relapse, long-term disease-modifying therapies (DMTs) and symptomatic treatment, relieving particular neurological symptoms [152]. Although there is already a wide range of DMTs that aim to reduce the autoreactive inflammatory response by targeting immunological pathways, they are only partially effective. These drugs modify and allow control of the active course of MS, but they have little or no potential to stop the disease permanently or reverse MS-related damage to the CNS. Furthermore, the majority of therapeutic agents target mainly immune-mediated active demyelination, while only a few drugs demonstrate some neuroprotective effects or are supposed to promote remyelination [153]. Recent progress in identifying SL properties and their role in the background of MS opens up a perspective in the design of novel therapeutic options, which would cover a wider extent of the MS pathophysiology.

With regard to immune-mediated activity, the endogenous myelin-derived acetylated β-GalCer ligands as well as synthetic α-GalCer ligand were used as stimulators of iNKT cells in MS patients, with subsequent diminished responsiveness of these regulatory cells observed [31]. Recognizing this phenomenon of GSL ligand-driven anergy and the functional consequences of the lipid–CD1d interaction may contribute to designing selective lipid antigen-specific therapeutics [138].

Targeting altered ganglioside metabolism [75,77] may represent another opportunity for a novel therapeutic approach in MS. An increased level of GM1 was found to correlate with enhanced susceptibility to an animal model of MS-EAE, while its binding to the endogenous lectin galectin-1 (Gal-1) ameliorated EAE symptoms [154]. Gal-1 seems an appealing candidate for the modulation of GM1 upregulation and enhanced autoimmune response. Furthermore, exogenous GM1 was demonstrated to reduce neuronal injury and neurodegeneration in animal models, and this effect reached beyond restoring normal levels of endogenous gangliosides. The suggested mode of action involved the modulation of various T cell effector functions [155] and suppression of pro-inflammatory Th1 cells’ activity [156]. Studies on EAE [155,156,157] have shown that the administration of brain ganglioside mixtures considerably reduces mortality, prolongs the lifespan and reduces the severity of clinical scores, roughly proportional to disease severity [157]. Beneficial clinical effects of these mixtures have been also reported in stroke and neurodegenerative disorders [3,72,158,159,160]. Thus, ganglioside-based therapies are likely to combine anti-inflammatory and neurotrophic effects.

Examples of complex SL-based agents, considered for potential therapeutic use in MS because of their effects on inflammation and degeneration in the CNS, are GluCer synthase inhibitors (PDMP, miglustat) and sGalCer. LacCer synthesis by β-1,4-galactosyltransferase 6 (B4GALT6) in astrocytes was shown to recruit and activate the CNS infiltrates (monocytes and microglia). The inhibition of LacCer synthesis by PDMP resulted in the suppression of local CNS innate immunity and neurodegeneration in EAE [91]. The proposed mechanism that promotes neuroinflammation and neurodegeneration in EAE includes the activation of cytosolic phospholipase A2 → a mitochondrial anti-viral signaling protein → NF-κB pathway [161]. Miglustat was found to inhibit this pro-inflammatory signaling pathway and suppress the CNS injury in a chronic progressive type of EAE [161]. Thus, PDMP [91,162] and miglustat [161,163] seem to be good candidates for therapeutic use in progressive MS, still not sufficiently addressed by available treatment strategies.

Another potential target for therapeutic options might be associated with impaired Cer metabolism, which is considered an important element of MS pathology [90,92,95,97,151]. Modulation of Cer metabolism could occur on many levels, including its de novo biosynthesis, SM hydrolysis and GSL breakdown, besides the re-acylation of Sph expression [90,164,165]. Our ex vivo studies could suggest that the increased Cer level in MS may come from the active dhCer→Cer pathway (de novo synthesis) in the case of Ac-MS or the active SM→Cer pathway (SM hydrolysis) in the case of In-MS [90]. Moreover, this investigation indicated C1P as a new potential biomarker of the progressive phase of MS. Thus, a novel therapeutic approach in this field might be developed [90].

The beneficial effect of ganglioside-based agents upon MS-related CNS damage might comprise also their potential to stimulate myelin repair. The contribution of GM1 to lipid raft formation may promote their stabilization and association with relevant proteins (e.g., Nf-155) and eventually prevent myelin sheath damage [102]. Administration of exogenous GM3, GM4 or GD1a was shown to induce the proliferation and differentiation of oligodendrocytes and maturation of their precursors [166,167,168], which resulted in initiating remyelination in in vitro and in vivo conditions [166]. Furthermore, sGalCer was also demonstrated to stimulate oligodendrocyte precursor differentiation, induced by laminin-2 binding [169].

Interesting observations concerning the possibilities of myelin repair were reported in studies on EVs containing SLs (e.g., S1P, GalCer and sGalCer) [170,171]. EV-associated SLs were suggested to have a dual effect on oligodendrocytes (affecting the balance between their apoptosis and maturation), as well as to boost the migration of oligodendrocyte precursors to the sites of demyelination, where they could stimulate repair processes. An intriguing approach proposed in another study [172] included the intranasal administration of EVs that contained a mixture of lipids, proteins and nucleic acids resembling the secretome of mesenchymal/parental stem cells. It is worth highlighting that these findings indicate a relevant role of SLs both as active agents and components of nano-carriers. Lipid-based EVs could be more effective in bypassing the BBB and reliably delivering a wide range of drugs or potentially active molecules to the affected structures of the CNS [65,173].

## 5. Conclusions and Future Directions

Despite recent progress in understanding the factors involved in the pathogenesis of MS and management of the disease, there are still some open questions worth further exploration.

The unique structural and functional features of SLs (especially gangliosides) offer new additions to the still incomplete view of these compounds, which have been referred to as a “*factotum of nature*” [58]. Recent studies suggest that SLs may represent key factors in the MS background in many aspects, including alterations of SL metabolic pathways, their role as putative antigens responsible for initiating the autoimmune response or modulators of iNKT cells’ regulatory features, as well as their contribution to the stability and functioning of intracellular lipid rafts. Further exploration seems necessary to clarify why particular SL-associated pathways become affected in MS, which of them are more specific for immune-mediated inflammation or neurodegeneration in the CNS and whether serum or CSF levels of gangliosides and anti-ganglioside antibodies might be used as biomarkers of disease activity and progression. Considering these challenges, SLs may represent an underexploited opportunity for further investigation in MS.

Intriguing future prospects of novel therapeutic options in MS are associated with SLs’ potential for myelin repair and neuroprotective effects, which have not yet been addressed in the available treatment strategies. The implications of the novel findings discussed in this review encourage the further development of SL-based investigations in the field of MS.

## Figures and Tables

**Table 1:** Ganglioside alterations in MS.

Ganglioside Subspecies	Altered In/Compared To	Specimen	References
GM1↓, GM2↓, Complete loss of GM4, GD2↑, GD3↑, polysialogangliosides↑	Chronic MS/WM of MS	Plaque	[75]
GM1 and GD1a expressed preferentially on oligodendroctyte precurors	Chronic MS/NAWM	Plaque	[76]
GT1b expressed selectively on astrocytes and oligodendroctyte precurors	Chronic MS/OND	Plaque	[76]
GM4↓, GM1↓, GD1b↓, GQ1b↓ GM3↑, GD3↑, GM2↑, GT1b↓	MS/nCNS	Spinal cord	[77]
GM1↑, GD1a↑, GT1b↓	chronic relapsing EAE/Lewis rats inoculated without myelin and/or not inoculated at all	Brain and spinal cord	[78]
GM3↑, GM1↑	MS/non-MS	CSF	[79]
GM1↑, GD3↑, sulfated glucuronyl paragloboside↑	MS/OND	CSF	[80]
Total gangliosides↓	MS/HS	Plasma	[81]
Total gangliosides↑ GM3↑, GD3↑	RRMS in remission/HS	Serum	[82]
GM1↑, GD1a↑, GM3↓	First attack of RRMS/HS	Serum	[83]
GM1↓, GD1a↑	Long duration of RRMS in relapse phase/HS	Serum	[83]
Total gangliosides↑	RRMS in remission/HS	PBMCs	[82]

The alterations are shown as colored arrows: increase in red and decrease in green. Abbreviations: CSF—cerebrospinal fluid, HS—healthy subjects, MS—multiple sclerosis, NAWM—normal appearing white matter, nCNS—normal central nervous system, OND—other neurological disease, PBMC—peripheral blood mononuclear cell, RRMS—relapsing–remitting MS.

**Table 2:** SL alterations in MS.

SL Subspecies *	Altered In/Compared To	Specimen	References
GalCer/sGalCer ratio↓	MS/nCNS	NAWM	[84]
Total sGalCer↓	MS/nCNS	NAWM	[85]
C18:0-Cer↓, C20:0-Cer↓, C22:0-SM↓, C24:0-SM↓	Ac-MS and In-MS/nCNS	NAWM	[87]
Total Cer↓, Sph↑, S1P↓, C16:0/C24:0-Cer ratio↑, C18:0/C24:0-Cer ratio↑	MS/nCNS and WM of AD	NAWM	[88]
C20:0-Cer↓, C22:0-Cer↓, C16:0-SM↓	Ac-MS and In-MS/nCNS	NAGM	[87]
C18:0-Cer↓, C20:0-Cer↓, C20:0-SM↓, C22:0-SM↓	Ac-MS/nCNS	NAGM	[87]
Total sGalCer↓, h24:0:0-sGalCer↑	MS/nCNS	Plaque	[85]
Total Cer↑	MS/X-adrenoleukodystrophy	Plaque	[86]
Total Cer↓, Sph↑, S1P↓, C16:0/C24:0-Cer ratio↑, C18:0/C24:0-Cer ratio↑	MS/nCNS, WM of AD	Plaque	[88]
C18:0-Cer↑	Ac-MS/nCNS	Plaque	[89]
C18:0-SM↓, C18:1-SM↓, C24-SM↓, C24:1-SM↓	In-MS/nCNS and In-MS/Ac-MS	Plaque	[90]
C16:0-HexCer↑, C18:0-HexCer↑, C18:1-HexCer↑, C24:0-HexCer↑, C24:1-HexCer↑	In-MS/nCNS and In-MS/Ac-MS	Plaque	[90]
C16:0-C1P↑, C18:0-C1P↑, C18:1-C1P↑, C24:0-C1P↑, C24:1-C1P↑	In-MS/nCNS and In-MS/Ac-MS	Plaque	[90]
Total LacCer↑	MS/WM of MS	Plaque	[91]
C16:0-Cer↑, C24:0-Cer↑, C16:0-HexCer↑	MS/ONDs	CSF	[92]
C16:0-HexCer↑	MS/ONDs	CSF	[93]
sGalCer↑	MS/nCNS	CSF	[94]
C16:0-Cer↑, C22:1-Cer↑, C24:1-HexCer↑, C24:1-LacCer↑, C22:0-LacCer↑, C20:0-dhCer↑, C24:0-dhCer↑, C16:1-HexCer↓, C20:1-LacCer↓, C26:0-dh-HexCer↓	RRMS and PPMS and SPMS/HS	Serum	[95]
C20:0-Cer↑, C20:1-Cer↑, C26:1-Cer↑, C20:1-HexCer↓, C22:1-HexCer↑, C16:0-HexCer↑, C18:0-HexCer↑, C20:0-HexCer↑, C22:0-HexCer↑, C26:0-HexCer↑, C16:1-LacCer↑, C16:0-LacCer↑, C22:0-dh-HexCer↑	Progressive MS/HS	Serum	[95]
C18:0-sGalCer↑, C24:1-sGalCer↑	RRMS/ RRMS with higher disability	Plasma	[96]
C16:0-Cer↑, C24:1-Cer↑, C16:0-GluCer↑, C24:1-GluCer↑, C16:0-LacCer↓	MS/HS	Plasma	[97]
C20:0-HexCer↑, C14:0-SM↓ C18:2-LPA↓	PPMS/HS	Plasma	[98]
C24:0-Cer↓, C16:0-LacCer↓	MS/HS	WBC	[97]

All listed SLs or dhCers are based on sphingosine (d18:1) or sphinganine (d18:0) backbones, respectively. The alterations are shown as colored arrows: increase in red and decrease in green. * SLs that contain N-acetylneuraminic acid have not been included in this table. They have been presented separately; see Table 1. Abbreviations: Ac-MS—chronic active multiple sclerosis, AD—Alzheimer’s disease, C1P—ceramide 1-phosphate, Cer—ceramide, CSF—cerebrospinal fluid, dhCer—dihydroceramide, GluCer—glucosylceramide, GM—grey matter, HexCer—hexosylceramide, HS— healthy subjects, In-MS—chronic inactive multiple sclerosis, LacCer—lactosylceramide, LPA—lysophospatidic acid, MS—multiple sclerosis, NAWM—normal appearing white matter, nCNS—normal central nervous system, OND—other neurological disease, PPMS—primary progressive MS, RRMS—relapsing–remitting MS, S1P—sphigosine 1-phosphate, SM—sphingomyelin, Sph—sphingosine, SPMS—secondary progressive MS, WBC—white blood cells, WM—white matter. The table was adopted from reference [99] and modified.

**Table 3:** Anti-ganglioside antibodies in MS.

Name of Antibodies against Gangliosides	Isotype(s)	Specimen	Percentage of Positive Patients/MS Subtype(s)	References
Anti-GM1	IgG and IgM	Serum	20%/SPMS	[109]
Anti-GM1	IgM	Serum	23.7%/n.d.	[110]
Anti-GM1	IgM	Serum	30%/n.d.	[111]
Anti-GM1	IgG	Serum	37.8%/RRMS	[112]
Anti-GM1	IgG and IgM	Serum	38%/n.d.	[113]
Anti-GM1	IgG	Serum	n.d./RRMS and SPMS	[114]
Anti-GM1	IgM	Serum	75%/n.d.	[115]
Anti-asialo-GM1	IgM	Serum	13.6%/n.d.	[110]
Anti-asialo-GM2	IgG and IgM	Serum	23.8%/n.d.	[113]
Anti-GD1a	IgG and IgM	Serum	33.3%/n.d.	[113]
Anti-GD1a	IgG	Serum	23%/n.d.	[116]
Anti-GD1a	IgG	Serum	40%/malignant MS	[116]
Anti-GD1a	IgG	Serum	6%/benign MS	[116]
Anti-GD1b	IgM	Serum	57%/n.d.	[115]
Anti-GM3	IgM	Serum	2.9%/RRMS	[117]
Anti-GM3	IgM	Serum	42.9%/SPMS	[117]
Anti-GM3	IgM	Serum	56.3%/PPMS	[117]
Anti-GQ1b	IgM	Serum	29%/n.d.	[115]
Anti-GD2-like	IgM	Serum	30%/n.d.	[118]
Anti-GD1a and GD3	IgM	Serum	Below 10%/n.d.	[118]
Anti-GM4	IgG	CSF	n.d./n.d.	[119]
Anti-GD1a	IgG	CSF	13%/n.d	[116]

Abbreviations: CSF—cerebrospinal fluid, GalCer—galactosylceramide, MS—multiple sclerosis, n.d.—not determined, PPMS—primary progressive MS, RRMS—relapsing–remitting MS, SPMS—secondary progressive MS.

## Data Availability

Not applicable.

## Abbreviations

Ac-MS	chronic active multiple sclerosis
AD	Alzheimer’s disease
BBB	blood–brain barrier
C1P	ceramide 1-phosphate
Cer	ceramide
CNS	central nervous system
CSF	cerebrospinal fluid
dhCer	dihydroceramide
DMT	disease modifying therapy
EAE	experimental autoimmune encephalomyelitis
EV	extracellular vesicle
FMC	fast migrating cerebroside
GalCer	galactosylcermide
GM	grey matter
GSL	glycosphingolipids
HexCer	hexosylceramide
HS	healthy subjects
iNKT	invariant natural killer T cells
In-MS	chronic inactive multiple sclerosis
INT	internode
JPX	juxtaparanode
LacCer	lactosylceramide
LPA	lysophosphatidic acid
MS	multiple sclerosis
MV	microvesicle
NAWM	normal appearing white matter
nCNS	normal central nervous system
NeuAc	N-acetylneuraminic acid
Nf	neurofascin
NKT cells	natural killer T cells
OND	other neurological disease
PN	paranode
PPMS	primary progressive multiple sclerosis
RRMS	relapsing–remitting multiple sclerosis
S1P	sphingosine 1-phosphate
SL	sphingolipid
SM	sphingomyelin
Sph	sphingosine
SPMS	secondary progressive multiple sclerosis
Th	T helper
WBC	white blood cell
WM	white matter
